# Dynamic observer: ion channel measurement beyond voltage clamp

**DOI:** 10.1186/1471-2202-12-S1-P238

**Published:** 2011-07-18

**Authors:** Damien Drix, Thomas Nowotny

**Affiliations:** 1 School of Informatics, University, University of Sussex, Brighton BN1 9QJ, UK; 2 School of Informatics and School of Life Sciences, University of Sussex, Brighton BN1 9QJ, UK

## 

To date, the gold standard for characterizing neurons and assessing the action of drugs on them are voltage clamp protocols in patch clamp recordings [1]. However, it is now clear that the classical procedure, where measurements performed using constant voltage steps and channel blockers are averaged over several cells, does not typically allow the construction of accurate Hodgkin-Huxley type models [2]. Here we propose to go beyond the classical procedure and rely on optimized stimulation patterns to isolate the effect of different ion channels.

We represent stimulation patterns as clamped cubic splines defined by a number of support points. Cubic splines can approximate steps or sinusoids, as well as arbitrary shapes; clamped splines avoid discontinuities around the endpoints. To measure the degree to which a given pattern can isolate the contribution of one channel, a reference neuron is detuned in two different ways. The first detuned neuron has one set of parameters increased by a certain factor, while in the second a different set of parameters is similarly detuned. The separation power of a stimulation pattern for these two sets of parameters is then defined as the ratio of the divergence factors of the two detuned neurons, which are defined as the sum of squared errors between the reference and detuned trans-membrane currents. Optimal patterns are constructed by adjusting the support points to maximize this ratio, typically through a combination of a genetic algorithm for exploring the search space, and gradient descent for fine-tuning.

**Figure 1:**
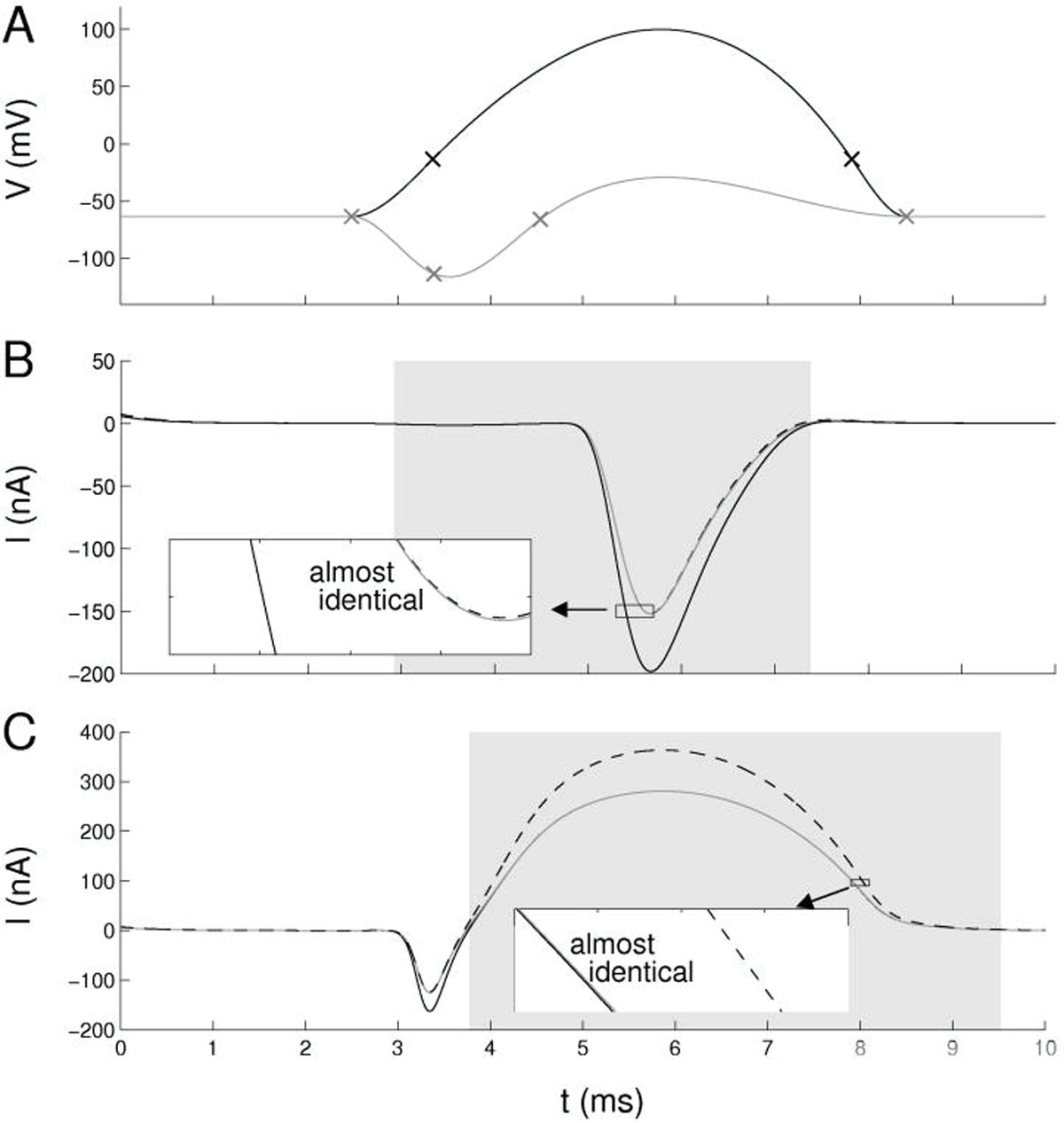
Optimized wave-forms and corresponding current measurements on a full HH model [3]. **A.** Voltage waveforms parameterized as clamped cubic splines with 4 support points (markers), one optimized to highlight the influence of g_Kd_ (black) the other to highlight g_Na_ (grey). **B.** The trans-membrane current when clamped to the grey input in three conditions: in control (grey), for increased g_Kd_ (dashed) and for increased g_Na_ (solid). **C.** Same as B) but for the black voltage control signal. The regions of interest (grey areas) were optimized to contain data where g_Na_ matters most (B) or g_Kd_ matters most (C). The deviation due to upregulation of the respective other parameter is minimal (see insets).

We show that for many parameters pairs in a Hodgkin-Huxley model, there are stimulation patterns which yield a clear dissociation between the effects of the selected parameters. Figure 1 shows a typical dissociation between g_Na_ and g_Kd_, but similar results have been obtained with other parameters including the membrane capacitance and reversal potentials. Some groups of parameters, however, are more difficult to separate.

To conclude, there clearly is a potential for isolating the effect of various parameters using optimized voltage clamp stimulation patterns. The offline process presented here is a first step towards a fully-automated online fitting method capable of extracting a model from a single cell in a patch clamp protocol. Such a method will be able to select the most informative stimulation pattern at any point of the fitting process and thus work incrementally to refine the fitting of these parameters which are less clearly dissociable from the others.
